# Integrated Metabolomics Assessment of Human Dried Blood Spots and Urine Strips

**DOI:** 10.3390/metabo7030035

**Published:** 2017-07-15

**Authors:** Jeremy Drolet, Vladimir Tolstikov, Brian A. Williams, Bennett P. Greenwood, Collin Hill, Vivek K. Vishnudas, Rangaprasad Sarangarajan, Niven R. Narain, Michael A. Kiebish

**Affiliations:** BERG, 500 Old Connecticut Path, Bldg. B, Framingham, MA 01701, USA; Jeremy_Drolet@waters.com (J.D.); brian.williams@berghealth.com (B.A.W.); bennett.greenwood@berghealth.com (B.P.G.); collin.hill@berghealth.com (C.H.); vivek.vishnudas@berghealth.com (V.K.V.); Rangaprasad.Sarangarajan@Berghealth.com (R.S.); Niven.Narain@Berghealth.com (N.R.N.); Michael.Kiebish@Berghealth.com (M.A.K.)

**Keywords:** human, dried blood spot (DBS), dried urine strip (DUS), metabolomics, chromatography, mass spectrometry

## Abstract

(1) Background: Interest in the application of metabolomics toward clinical diagnostics development and population health monitoring has grown significantly in recent years. In spite of several advances in analytical and computational tools, obtaining a sufficient number of samples from patients remains an obstacle. The dried blood spot (DBS) and dried urine strip (DUS) methodologies are a minimally invasive sample collection method allowing for the relative simplicity of sample collection and minimal cost. (2) Methods: In the current report, we compared results of targeted metabolomics analyses of four types of human blood sample collection methods (with and without DBS) and two types of urine sample collection (DUS and urine) across several parameters including the metabolite coverage of each matrix and the sample stability for DBS/DUS using commercially available Whatman 903TM paper. The DBS/DUS metabolomics protocols were further applied to examine the temporal metabolite level fluctuations within hours and days of sample collection. (3) Results: Several hundred polar metabolites were monitored using DBS/DUS. Temporal analysis of the polar metabolites at various times of the day and across days identified several species that fluctuate as a function of day and time. In addition, a subset of metabolites were identified to be significantly altered across hours within a day and within successive days of the week. (4) Conclusion: A comprehensive DBS/DUS metabolomics protocol was developed for human blood and urine analyses. The described methodology demonstrates the potential for enabling patients to contribute to the expanding bioanalytical demands of precision medicine and population health studies.

## 1. Introduction

Dried blood spot testing (DBS) has been used for screening for inborn errors of metabolism for over 50 years [1,2]. Every newborn in most developed countries is screened via DBS sampling [2,3]. DBS offers several advantages over conventional whole blood, plasma, or serum sample collection [4,5]. For example, DBS sample collection is minimally invasive and easy to perform (e.g., finger prick for adults, rather than venous puncture, and heel prick for infants). Moreover, in the case of adults, blood sampling can be successfully performed at home by patients themselves after minimal training. In addition, a low volume of blood (30 µL–100 µL per spot) is needed to spot onto filter paper compared to a minimum of 0.5 mL of blood in venous sampling. Air-dried DBS samples can be shipped by mail to bio-analytical and clinical laboratories without the expense and infrastructure associated with freezing a sample or maintaining it at a stable temperature prior to analysis. 

**Figure 1:**
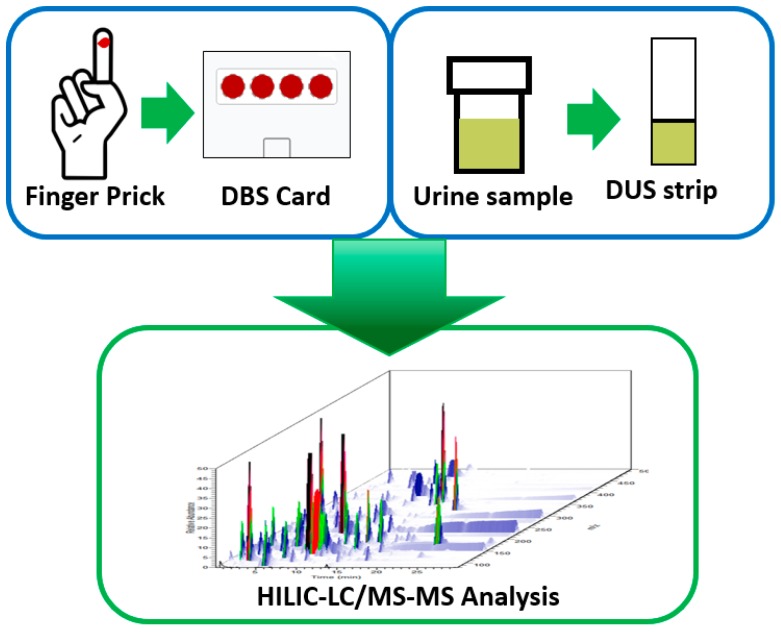
Schematic of human metabolome analysis using DBS/DUS sampling protocols.

The application of DBS has been further developed for therapeutic drug monitoring [6,7,8,9], pharmacokinetics [10], genomics [11], proteomics [12,13], lipidomics [14,15,16] and metabolomics studies [17,18,19,20,21,22,23,24]. Non-targeted and targeted metabolomics analyses have been performed using DBS [17,18,19,20,21,24]. Additionally, metabolic biomarker measurements from samples captured via DBS have also been reported [21,22]. Another much less utilized, but very similar sampling approach is the analysis of dried urine on filter paper, called dried urine strips (DUS). This approach has previously been used in other applications such as creatinine testing [25,26], HPV (human papillomavirus) testing [27], and metabolomics analysis [28,29]. Herein, we report on a DBS/DUS-HILIC-LC-MS/MS protocol development for analysis of the human blood and urine metabolome (Figure 1). We also highlight that the DBS/DUS-GC-MS blank and matrix reveal background carrier-induced interferences, which may impact sample analysis. 

In this study, we used mass spectrometry to measure the metabolite, separated by gas and liquid chromatography, and utilized previously described GC-MS and HILIC-LC-MS/MS procedures for polar metabolites analysis [30,31]. More than three hundred polar metabolites were detected and measured from a single DBS/DUS sample. We explored the metabolome profiles from four different blood matrices: (1) DBS from a finger prick, (2) DBS from whole blood (through venous sampling), (3) liquid whole blood (through venous sampling), (4) liquid plasma and two urine matrices; as well as (5) DUS from liquid urine, and (6) liquid urine. DBS/DUS metabolite stability at various storage temperatures and time points was also assessed. The DBS/DUS-HILIC-LC-MS/MS workflow development demonstrated the possibility of utilizing this metabolomics tool for the monitoring of population health and facilitating the creation of precision medicine datasets for diverse disease states and interventions [32,33,34,35]. 

## 2. Results 

### 2.1. HILIC-LC-MS/MS and GC-MS Analyses of Different Blood Matrix Samples Highlight Differential Metabolite Detection from Different Sample Preparation Techniques

In order to investigate the potential difference between DBS samples prepared from different matrices, four types of sample were assessed in this study: (1) DBS prepared with finger pricked blood (capillary drawn), (2) DBS prepared with whole blood (venous drawn), (3) DBS prepared with plasma, and (4) liquid plasma. All DBS samples were spotted onto Whatman 903TM filter paper cards, and all samples, including plasma, were extracted as previously described [30]. More than 300 polar metabolites were detected in each blood sample. As expected, the majority of metabolites detected were present in each sample regardless of preparation. However, we observed differential metabolite detection specific to each matrix used (less than 15% of the detected metabolites) (Table S1, Supplementary Materials). We therefore investigated the detection of potential interfering contaminants from the Whatman 903TM filter paper cards. We analyzed the DBS extracts of samples prepared with a blank card using our GC-MS protocols [30]. We found that there was no contamination originating from the filter paper cards that could be detected with GC-MS (Figure S1, Supplementary Materials). 

### 2.2. HILIC-LC-MS/MS and GC-MS Analyses of Different Urine Matrix Samples Demonstrate Differential Metabolite Detection Dependent on Sample Preparation Techniques

In order to investigate the potential differences between samples prepared from human urine matrices, two urine matrices were assessed in this study: (1) liquid urine and (2) DUS prepared with liquid urine. All DUS samples were collected by dipping a strip cut from a Whatman 903TM filter paper card into a collected urine sample. Samples were extracted as previously described [30]. More than 300 polar metabolites were detected from both liquid urine and DUS samples. However, we observed differential detection of metabolites and paper carrier interferences for DUS (Table S2, Supplementary Materials). Table S2 depicts a list of human urine metabolites (less than 12% of the detected metabolites) from which detection was impacted by the sample preparation protocol applied or by the sample carrier interference.

### 2.3. Temperature and Duration of Sample Storage Impacts Subsequent Metabolite Analysis in DBS and DUS 

**Figure 2:**
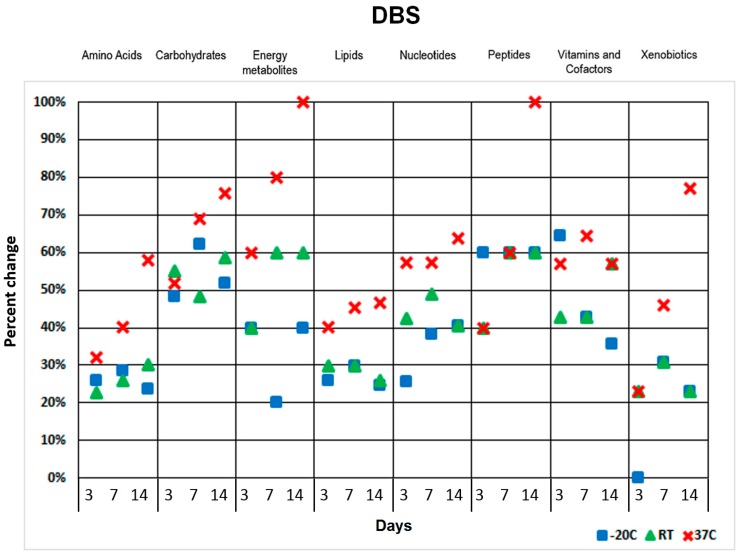
Two weeks DBS storage stability results for detected human blood metabolite classes assigned by participation in major pathways. Y axis represents percent change calculated using the mean of triplicate measurements for a metabolite class. Each column shows three measurement points at 3, 7 and 14 days (from left to right).

One of the advantages of DBS and DUS probes is improved sample stability over a wide range of temperatures during storage and transportation [4,5,9]. We stored both DBS and DUS samples at −20 °C, room temperature (RT), and 37 °C for three days, one week, and two weeks. Samples were analyzed using HILIC-LC-MS/MS protocols as described previously [30]. The stability was determined by the comparison of the metabolite levels of each chemical class of the samples with the control samples (day 0). Significant variations in several metabolite classes were observed between time points at higher storage temperatures compared with storage at −20 °C (Figure 2). The metabolite group compilation shown was based on the concept of metabolic pathways, with related metabolites grouped together.

**Figure 3:**
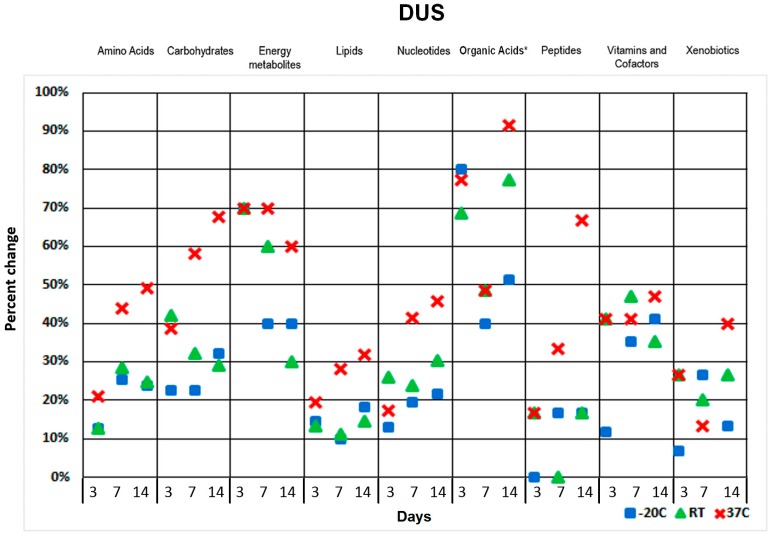
Two weeks DUS storage stability results for detected human urine metabolite classes. *—organic acids were measured with GC-MS protocols. Y axis represents percent change calculated using mean of triplicate measurements for a metabolite class. Each column shows three measurement points at 3, 7 and 14 days (from left to right).

Urine contains a variety of metabolism products removed from the circulation. A significant portion of these waste products are organic acids. In order to assess the variance of organic acids in urine we applied GC-MS protocols allowing excellent organic acid isomer resolution. For most of the selected metabolite classes, the relative change was less than 30–40% when samples were stored at −20 °C or even at room temperature for up to one week, except for energy metabolites in DBS/DUS and organic acids in DUS (Figure 3). 

By contrast, storing at 37 °C impacted stability for the vast majority of metabolites even at short durations of storage. An overview of the detected metabolite level variations observed during the short-term DBS stability study is displayed in a heat map (Figure S2). The output of hierarchical clustering data generated with two-way ANOVA (analysis of variance) analysis illustrated sample deterioration due to extended storage (two weeks) at a higher temperature (37 °C) (Figure S2). 

### 2.4. DBS/DUS Inter-Day Variation Study

In order to assess the day to day variation of the human blood and urine metabolome when using DBS/DUS probes, healthy volunteers were recruited and DBS/DUS samples were collected over the course of five successive mornings after overnight fasting. Samples were analyzed using HILIC-LC-MS/MS protocols as previously described [30].

**Figure 4:**
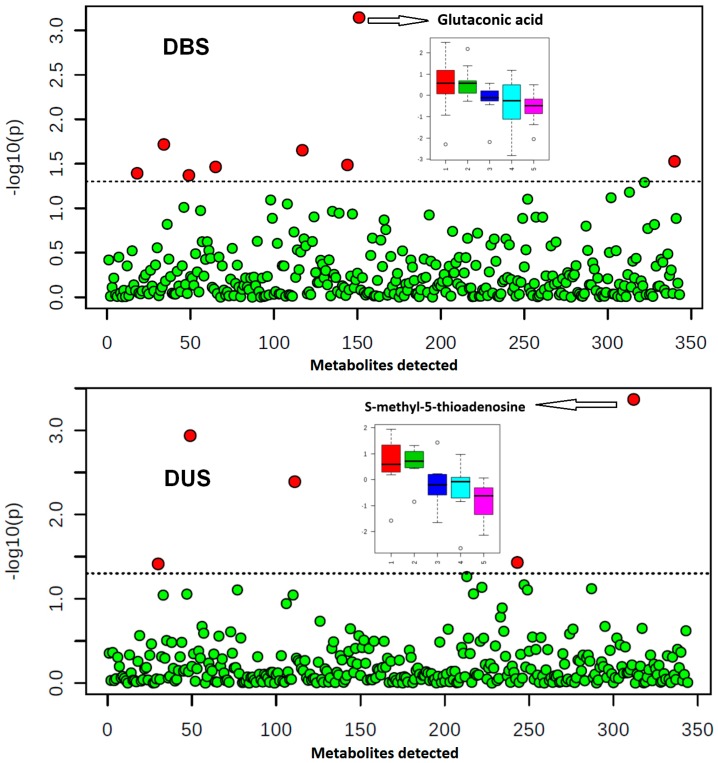
Temporal changes of metabolomes over days (morning fasting sample collection). Upper panel: DBS one-way ANOVA analysis results (subject = 16, days = 5, p value threshold 0.05). Lower panel: DUS one-way ANOVA analysis results (subject = 10, days = 5, p value threshold 0.05). X axis shows total number of metabolites detected. Red circles illustrate metabolites which levels showed statistically significant differences. Box plots depict the most impacted metabolite temporal changes at time points 1–5 (corresponding to five consecutive days).

Results of the one-way ANOVA analysis suggest a low level of inter-day variability for DBS and DUS matrices prepared and analyzed in-house (Figure 4). The percent change does not exceed 2.5% for either DBS or DUS. The most variable metabolites are depicted in box plots illustrating daily variability.

### 2.5. DBS Intra-Day Variation Study

**Figure 5:**
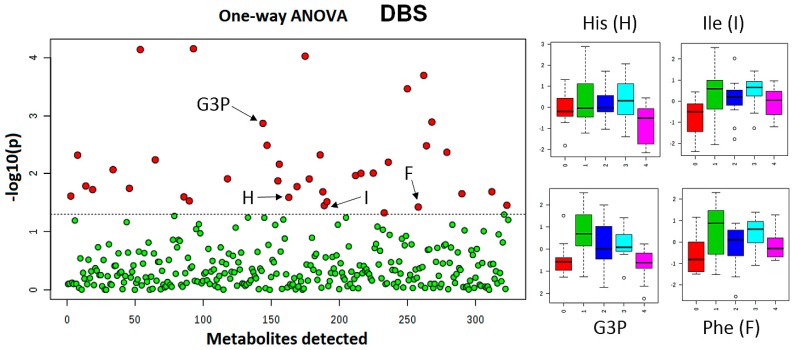
Intra-day variation of blood metabolome. One-way ANOVA analysis results (subject = 12, days = 1, p value threshold 0.05) for DBS samples. Left panel: X axis presents total number of metabolites detected. Red circles illustrate metabolites which levels showed statistically significant differences. Right panel: Box plots depict selected metabolites temporal changes at time points 0–4 (corresponding to fasting 7 AM, 10 AM, 1 PM, 4 PM, and 7 PM). Y axis shows the normalized relative abundance.

To study the potential temporal human blood metabolome fluctuation, 12 volunteers were recruited and DBS collection was performed at five time points (fasting 7 AM, 10 AM, 1 PM, 4 PM, and 7 PM). Samples were collected, extracted, and analyzed using HILIC-LC-MS/MS protocols as described previously [30]. The −log10 (*p*-value) was plotted against each individual metabolite measured levels (Figure 5) to identify the most dynamic metabolite molecules that were altered over the course of the day.

Box plots in Figure 5 illustrate the pattern of selected essential amino acids and glyceraldehyde-3-phosphate levels (G3P) variations in the blood of the volunteers participating in this study. This pattern depicts elevated levels of essential amino acids in the blood stream provided by food intake at various time points during the day. A similar trend was observed for G3P levels variations. G3P is known as an important metabolic intermediate in both glycolysis and gluconeogenesis. 

**Figure 6:**
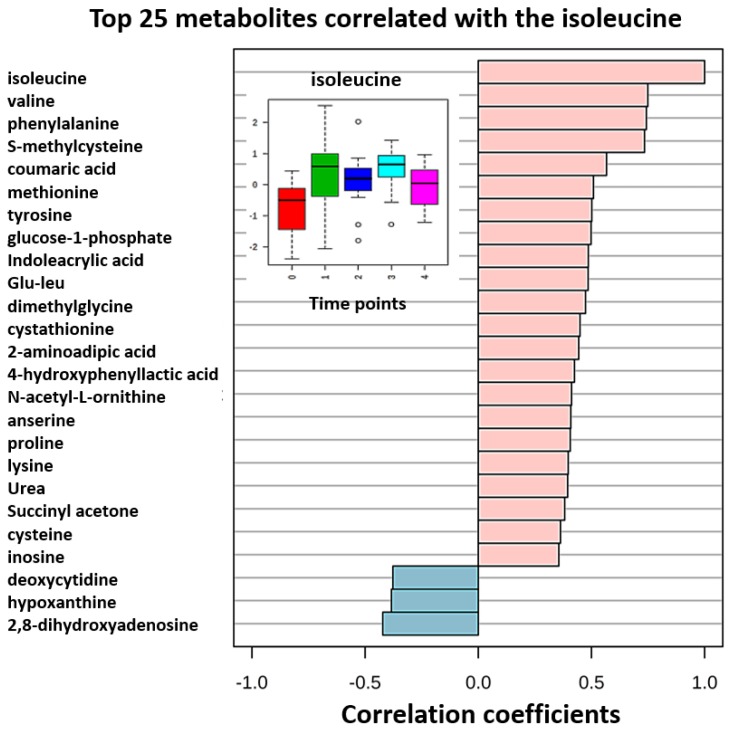
Top 25 metabolites correlating with isoleucine level patterns at time points 0–4 (corresponding to fasting 7 AM, 10 AM, 1 PM, 4 PM, and 7 PM), which correlated to intra-day variation in the blood metabolome analyzed with DBS protocols.

In order to confirm this observation and investigate further, a correlation analysis was performed (Figure 6). In this case we were looking for pattern correlations similar to the isoleucine levels pattern within a study day. Similar patterns were observed for phosphoryl choline and phosphoryl ethanolamine levels reflecting lipid metabolism variations impacted by food consumption and digestion.

**Figure 7:**
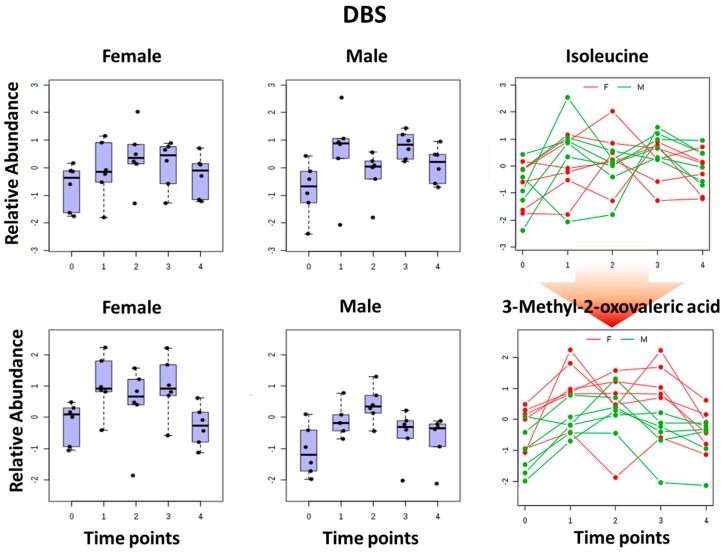
Two way ANOVA analysis results. Upper panel depicts isoleucine intra-day variations in the blood metabolome. Lower panel shows 3-methyl-2-oxovaleric acid intra-day variations in the blood metabolome. 3-methyl-2-oxovaleric acid is a downstream metabolite of isoleucine in humans (red arrow). Y axis shows normalized relative abundance. X axis illustrates intra-day time points corresponding to fasting, 7 AM, 10 AM, 1 PM, 4 PM, and 7 PM (0–4, from left to right).

We also performed a two-way ANOVA analysis with a focus on essential amino acids and their downstream metabolites. The results of this analysis in particular for isoleucine are shown in Figure 7. It was found that isoleucine and its downstream metabolite 3-methyl-2-oxovaleric acid are at different levels during the day for males and females.

## 3. Discussion

The DBS/DUS approach is a convenient way to collect blood/urine samples with several advantages over conventional collection methods. These methods have gained popularity in fields such as newborn screening [1,2], preclinical studies [17,18,19,20,21,22,23,24], and therapeutic drug monitoring [10]. DBS/DUS offers a high-throughput approach to the analysis of biofluid samples. Metabolomic analysis of biofluids, in particular blood and urine, allow for the analysis of particular metabolic perturbations that may be connected with an individual’s physiological, nutritional, and health status [5,8,21,32,33,34,35]. In the current study, we utilized the DBS/DUS capabilities of sample collection coupled with mass-spectrometry-based metabolomics analysis [30] to explore human blood and urine metabolomic profiling. We reported on the routine detection of more than 300 polar metabolites in a single DBS/DUS sample. Intact lipids and small molecules that belong to human biofluid lipidomes [14,15,16] were not included in the DBS/DUS applied analytical protocols in this study. We also investigated the stability of DBS and DUS samples in comparison to their liquid equivalent samples. We here reported on metabolites susceptible to deterioration during short-term storage at different temperatures. We found that the list of metabolites resistant to environmental impact shortens with the increase of the severity of environmental impact. We also found that DBS samples prepared with finger-pricked blood (capillary drawn) possess high stability and reproducibility when sampling is performed and processed in-house. 

The importance of multi-omics profiling has recently become a focus of developments dedicated to the analysis of molecular signatures for precision medicine applications. In particular, metabolomic analysis is an important component of these exciting developments [32,33,35]. Metabolomics, as an essential part of multi-omics platforms, can deliver valuable information illuminating the complexity of environmental influence and population phenotype, as well providing a wealth of information for patient stratification and the support of guided clinical trials [34,35]. Herein, we examined the compatibility of the DBS/DUS approach with conventional blood/urine matrices using mass-spectrometry-based metabolomics protocols. Three types of blood samples were taken and spotted onto DBS cards: capillary drawn (finger prick), venous drawn, and plasma (EDTA). DBS-originated extracts were compared with liquid plasma extracts using HILIC-LC-MS/MS protocols [30]. The target list designed for human biofluid analysis contains 428 endogenous analytes. As expected, a majority of expected polar metabolites were detected in each sample matrix. About 350 metabolites were detected from the blood samples. We discovered approximately 15% of the metabolites detected from DBS were lost in comparison to the liquid plasma analysis. Amongst these missing metabolites were ATP, 5-Methyl-THF, iso-citrate, and other phosphates. DUS was compared with liquid urine in the same manner. It was found that carrier interference and perhaps information loss impacted approximately 12% of metabolites detected using the DUS approach. Nucleosides, nucleotides, folates and sugar phosphates were found to be the most compromised entities in urine sample analysis. The metabolite loss in the DBS and DUS may be due to the air dying conditions employed in sample preparation. 

We also studied DBS/DUS short-term sample stability at a range of storage temperatures. It was found that DUS had fewer changes in metabolite levels compared with DBS under short-term storage conditions. Higher temperatures and longer storage time had the most impact on both blood and urine metabolome stability. Carbohydrates, energy metabolites, nucleotides and vitamins were found to be the most sensitive to environmental storage conditions in DBS. Carbohydrates, energy metabolites and organic acids were found to be strongly impacted in DUS under short-term storage conditions. DBS proved to be sensitive to even short-term storage with some metabolite classes altered even after only three days of storage. 

Using DBS and DUS, we investigated the inter-day and intra-day variations in healthy volunteers’ blood and urine. In this study, blood samples were processed the same day of collection, and the following day for urine collection, respectively. The metabolome profiles of individuals were consistent across days, which implies that fasted samples are quite stable across study days amongst different individuals, when collected and processed in-house. Few metabolite levels between DBS/DUS were found to be statistically different in blood and urine metabolomes over the study days. This may be reflective of changes in biological processes such as circadian rhythms (internal factors) and changes in diet over sample collection days (external factors). Observation of this high level of DBS/DUS stability during in-house sample collection and processing presents a unique opportunity for pre-clinical and clinical studies. Investigation of intra-day blood metabolome variations using DBS allowed us the opportunity to monitor biological events associated with the daytime systemic metabolomic processes in humans.

DBS sampling offers less invasive multiple blood collection procedures during a day at several time points in comparison to venous blood sampling. One-way ANOVA analysis revealed that approximately 11% of the monitored metabolites showed statistically significant variance within the day of collection. Essential amino acids were found amongst the highly variable species. This finding indicates that external factors, such as food consumption, have a profound impact on the participants’ metabolome profiles. Metabolic activity changes during fasting, postprandial, and post-absorptive periods. Blood levels of essential amino acids, energy metabolites and some BCAA metabolites follow this pattern. G3P or Glyceraldehyde 3-phosphate is a chemical compound that occurs as an intermediate in several central metabolic pathways, such as glycolysis, gluconeogenesis, tryptophan and thiamine biosynthesis. The second phase of glycolysis, the energy-yielding phase, creates the energy that is the product of glycolysis. Glyceraldehyde-3-phosphate dehydrogenase converts each three-carbon glyceraldehyde-3-phosphate produced during the energy-consuming phase into 1,3-bisphosphoglycerate. Observed variances in the samples collected illustrate the metabolic biological events captured at a particular time point associated with fasting, food uptake and digestion Amongst the top 25 metabolites that correlated with the isoleucine pattern over the course of the study day, other essential amino acids were found (Figure 6). Branched chain amino acids (BCAA) are essential amino acids whose carbon structure is marked by a branch point. 3-methyl-2-oxovaleric acid is a downstream metabolite of isoleucine in humans. Anserine is a dipeptide containing β-alanine and 1-methylhistidine, which can be found in the skeletal muscles and brains of mammals and birds, i.e., various foods. Dimethylglycine is a derivative of the amino acid glycine with the structural formula (CH₃)₂NCH₂COOH and can be found in grains. Samples collected at time point 0 (corresponding to fasting 7 AM time) contained the lowest amounts of essential amino since these substances were depleted overnight. According to postprandial detection throughout the day, these metabolites were delivered into the blood stream, showing highest levels at time points 1 (10 AM) and 3 (4 PM). These levels declined further as expected at time point 4 (7 PM). We also investigated if DBS-HILIC-LCMS/MS platform analysis could deliver information pertaining to potential gender differences in this experiment. Participants were a group of 16 healthy volunteers (eight male and eight female, mean age 32 ± 6 years). Two-way ANOVA analysis resulted in our finding differences in levels of essential amino acids and their downstream metabolites in males and females participating in this experiment. Figure 7 illustrates results of this analysis in particular for isoleucine metabolism. We found that isoleucine metabolism demonstrates different pace for males and females within a same day. Specifically, at time point 2 (1 PM) females have higher isoleucine levels than males and subsequently 3-methyl-2-oxovaleric acid levels are lower in females. This may indicate that males metabolize isoleucine faster than females at this time point. These findings illustrate the capability of the DBS-HILIC-LCMS/MS platform to deliver valuable biological information in pre-clinical and clinical capacities.

In this investigation, we found that the molecular species identified by the DBS/DUS-HILIC-LC-MS/MS platform have the potential to deliver valuable insight for the application of personal metabolome monitoring. This type of analysis could be further applied to diagnostic developments and possibly to diet and lifestyle recommendations. The challenges for developing this platform included the lack of extensive references for most molecular species using DBS/DUS for sampling and the possible interaction of analytes with the matrix of a DBS/DUS card, which were addressed by some of the experiments reported herein. There are several indications that DBS/DUS sampling combined with the mass-spectrometry-based analytical platforms could be applied to future large-scale population monitoring studies.

## 4. Materials and Methods 

### 4.1. Chemicals and Reagents

Deuterated phenylalanine (d8 phenylalanine) was purchased from Cambridge Isotope Laboratories (Tewksbury, MA, USA). LC/MS grade solvents were purchased from Fisher Scientific (Waltham, MA, USA) or VWR International (Radnor, PA, USA). Whatman 903TM filter paper cards, adjusted ACCU-CHEK lancets, and Uni-Core punch with ID 3mm were purchased separately from GE Healthcare (Westborough, MA, USA) and Roche Diagnostics (Indianapolis, IN, USA). 

### 4.2. Ethical Statement

All healthy volunteers participating in this study, gave their informed consent for inclusion before they participated in the study. Research use of the samples was conducted in accordance with the terms outlined within the informed consent form and the terms set forth therein and with the tenets of the Declaration of Helsinki. Diets and other physiological parameters were not controlled in this study.

### 4.3. Preparation of Dried Blood Spots 

For the blood matrix study, a total of 16 healthy volunteers (eight male and eight female) were included (mean age 32 ± 6 years). Participants fasted overnight (around 12 h) and venipuncture of a cubital vein was performed in the morning. Blood was drawn into a non-coated tube for whole blood, and into a K2 EDTA tube (BD) for blood plasma. For whole blood samples, tubes were mixed well by gentle inversion 10 times and whole blood was immediately spotted on Whatman 903TM filter paper cards. For plasma samples, the tubes were centrifuged at 1200 × *g* for 15 min at room temperature and the supernatant plasma were spotted onto Whatman 903TM filter paper cards. Concomitantly, blood via finger prick was taken and spotted onto Whatman 903TM filter paper cards. All samples were air dried for 3 h at room temperature (−22 °C) and collected using Uni-Core punch (ID 3 mm, GE Healthcare, Chicago, IL, USA) for extraction as described in Section 4.6. 

For the short-term DBS stability study, whole blood was drawn and immediately spotted onto Whatman 903TM filter paper cards. After samples were air dried completely for 3 h at room temperature, the paper cards were stored with desiccant at different conditions: 4 °C, room temperature, and 37 °C for three days, one week, and two weeks. Samples were used for extraction for the initial time point (T = 0 d), and subsequently sampled on days 3, 7 and 14 from each storage condition in triplicate. 

For the time course study, a total of 12 healthy volunteers (six male and six female) were included (mean age 31 ± 6 years). After finger pricks using a single-use safety lancet, the blood drop was directly applied onto Whatman 903TM filter paper cards at 7 AM (after overnight fasting), 10 AM, 1 PM (1 h after lunch), 4 PM, and 7 PM. The blood spots were air dried completely for 3 h at room temperature and stored at room temperature in a zip-closure bag with desiccant. Samples were collected and used for extraction for all time points in triplicate. 

For the DBS daily variation study, a total of 16 healthy volunteers (eight male and eight female) were included (mean age 32 ± 6 years). Finger prick blood samples were collected onto Whatman 903TM filter paper cards in the morning after overnight fasting over five consecutive days. The blood spots were air dried completely for 3 h at room temperature and stored at room temperature in a zip-closure bag with desiccant [14]. Samples were collected and used for extraction for all time points in triplicate.

### 4.4. Preparation of Dried Urine Strips 

For the urine matrix study, morning urine midstream samples of equal volume (15 mL) were collected from a total of three female and three male volunteers and the samples were pooled. Precut strips of Whatman 903 protein saver cards were briefly submerged in the resultant pooled urine and allowed to dry at room temperature for 3 h and collected using Uni-Core punch (ID 3 mm, GE Healthcare, Chicago, IL, USA) for extraction as described in Section 4.5.

For the short-term DUS stability study morning urine pooled sample described above was used. Precut strips of Whatman 903 protein saver cards were briefly submerged in the resultant pooled urine and allowed to dry at room temperature for 3 h. Samples were placed in sealed zip-closure bags with 4 g silica gel packets to prevent moisture intrusion and stored at −20 °C, room temperature, and 37 °C, respectively. Samples were used for extraction for the initial time point (T = 0 d), and subsequently sampled on days 3, 7 and 14 from each storage condition in triplicates.

For the DUS daily variation study, a total of 10 healthy volunteers (five male and five female) were included (mean age 32 ± 6 years). Precut strips of Whatman 903 protein saver cards were used in the morning after overnight fasting over five consecutive days. The strips were air dried completely for 3 h at room temperature and stored at room temperature in a zip-closure bag with desiccant. Samples were collected and used for extraction for all time points in triplicate.

### 4.5. HILIC-LC-MS/MS and GC-MS Analyses

HILIC-LC-MS/MS analyses were performed using the NEXERA XR UPLC system (Shimadzu, Columbia, MD, USA), coupled with the Triple Quad 5500 System (AB Sciex, Framingham, MA, USA) as described in Tolstikov et al., 2014 [30]. GC-MS analyses were performed as described in Tolstikov et al., 2014 [30] using an Agilent 7890B gas chromatograph (Agilent, Palo Alto, CA, USA) interfaced to a time-of-flight Pegasus HT mass spectrometer (Leco, St. Joseph, MI, USA). Automated injections were performed using an MPS2 programmable robotic multipurpose sampler (Gerstel, Muhlheim an der Ruhr, Germany). The GC system was fitted with a Gerstel temperature-programmed injector, cooled injection system (model CIS 4). An automated liner exchange (ALEX) (Gerstel, Muhlheim an der Ruhr, Germany) was used to eliminate cross-contamination from the sample matrix that was occurring between sample runs.

### 4.6. DBS and DUS Sample Preparation and Analysis 

Samples for HILIC-LC-MS/MS analysis were extracted by punching out four discs for DUS and two disks for DBS uniformly using a GE Healthcare Uni-Core punch with ID 3 mm and transferred to 0.5 mL Eppendorf tubes. To each tube, 75 µL of extraction solvent (3:3:2 isopropanol/acetonitrile/water) and 5 µL of internal standard solution (1 µg/mL d8-phenylalanine) were added. The samples were vortexed briefly, sonicated for two minutes, and allowed to stand at room temperature for 30 min. Samples were centrifuged at 14,000 RPM for five minutes. 50 µL of the resultant supernatant were collected for HILIC-LC-MS/MS analysis. Blank paper extracts were prepared using untreated paper cards and the same extraction protocol. Samples were analyzed as described [30].

Samples for GC-MS analysis were extracted by punching out four discs for DUS and two disks for DBS uniformly using a GE Healthcare Uni-Core punch with ID 3 mm and transferred to 0.5 mL Eppendorf tubes. To each tube, 50 µL of water were added. Samples were vortexed briefly and sonicated for two minutes. Samples were treated with 5 µL of urease enzyme solution (25 mg urease in 500 mL water) for 1 h at 37 °C. Following urease treatment, 400 µL of methanol were added and extraction was allowed to proceed for 1 h at −20 °C. Samples were then centrifuged for five minutes at 14,000 RPM. 200 µL of the clear supernatant were collected and dried overnight using SpeedVac Concentrator Savant DNA120 (ThermoFisher, Waltham, MA, USA) at 30 °C. Blank paper extracts were prepared using untreated paper cards and applying the same extraction protocol. Dried samples were derivatized and analyzed as described in Tolstikov et al., 2014 [30].

### 4.7. Plasma and Urine Sample Preparation and Analysis 

Plasma samples for HILIC-LC-MS/MS analysis were vortexed for 10 s and then aliquoted 75 µL into labeled 1.5 mL Eppendorf tubes. 450 µL of extraction solvent (3:3:2 isopropanol/acetonitrile/water) and 5 µL of internal standard solution (1 µg/mL d8-phenylalanine) were added. This was vortexed for five seconds and stored at −20 °C overnight. It was then centrifuged at 14000 RPM for 10 min at 4 °C. Clean supernatant was collected for HILIC-LC-MS/MS analysis. 200 µL of the clear supernatant were collected and dried overnight using SpeedVac Concentrator Savant DNA120 at 30 °C. Dried samples were derivatized and analyzed as previously described in [30].

Urine samples for HILIC-LC-MS/MS analysis were vortexed for 10 s and then aliquoted 175 µL into labeled 1.5 mL Eppendorf tubes. 175 µL of acetonitrile and 5 µL of internal standard solution (1 µg/mL d8-phenylalanine) were added. This was vortexed for five seconds and then centrifuged at 14.0× *g* for 10 min at 4 °C. Clean supernatant was collected for HILIC-LC-MS/MS analysis. 50 µL of neat urine samples for GC-MS analysis were treated with 5 µL of urease enzyme solution (25 mg urease in 500 mL water) for 1 h at 37 °C. Following urease treatment, 400 µL of methanol were added and extraction allowed to proceed for 1 h at −20 °C. Samples were then centrifuged for five minutes at 14,000 RPM. 200 µL of the clear supernatant were collected and dried overnight using SpeedVac Concentrator Savant DNA120 (ThermoFisher) at 30 °C. Dried samples were derivatized and analyzed as previously described [30].

### 4.8. Data Processing and Statistical Analysis

Metabolomic data was analyzed as previously described [30,31]. The results are expressed as mean ± standard deviation (SD). Metabolomics data processing and normalization was conducted using MetaboAnalyst 3.0. Sample normalization by reference was accomplished by means of recorded internal standard (d8-phenylalanine) levels. Alternatively, median normalization was applied. Normalized data was further log transformed and auto-scaled. Statistical analysis of metabolites was performed using one-way analysis of variance ANOVA. Temporal and two-factor data analysis including data overview, two-way ANOVA, and empirical Bayes time-series analysis for detecting distinctive temporal profiles was conducted where applicable. ANOVA-simultaneous component analysis (ASCA) was applied to identify major patterns associated with each experimental factor using MetaboAnalyst 3.0 [36].

## 5. Conclusions

Blood metabolome changes during fasting, postprandial and post-absorptive intra-day periods demonstrated dynamic changes for future utilization in preclinical and clinical studies, as well as for population based health studies. As a less invasive sampling method, DBS/DUS offers a simple collection protocol, and it is easy to store and transfer samples. Analysis of an individual’s blood and urine metabolome using this workflow provides a valuable approach for real-time health status monitoring that is critical in the era of precision medicine.

## Supplementary Materials

The following are available online at http://www.mdpi.com/2218-1989/7/3/35/s1, Table S1: Impacted human blood metabolites in different matrices, Table S2: Impacted human urine metabolites in DUS, Figure S1: GC-TOF-MS mass chromatograms, Figure S2: Hierarchical clustering analysis results of two weeks DBS storage stability study.
