# Supplementary material for: Integrated Metabolomics Assessment of Human Dried Blood Spots and Urine Strips
Source: Metabolites. 2017 Jul 15;7(3):35. doi: 10.3390/metabo7030035 (PMC5618320; doi:10.3390/metabo7030035)
Supplement: Supplementary file 1 [file metabolites-07-00035-s001.pdf]

Supplementary Materials: Integrated Metabolomics Assessment of Human Dried Blood Spots and Urine Strips

Jeremy Drolet, Vladimir Tolstikov *, Brian Williams, Bennett Greenwood, Collin Hill, Vivek K. Vishnudas, Rangaprasad Sarangarajan, Niven R. Narain and Michael A. Kiebish

Table S1. Impacted human blood metabolites in different matrixes.

Metabolite	DBS Capillary	DBS Venous	DBS Plasma	Liquid Plasma
1-Phosphatydyl-myo-inositol				•
2,3-Dihydroxybenzoic acid				•
2-Octandioyl-carnitine	•			•
2-Octendioyl-carnitine	•		•	•
3-Hydroxy-3-methylglutarylcarnitine	•	•		•
3-Hydroxysuberoylcarnitine				•
3-Ketosuberoylcarnitine				•
3-Phosphoglycerate	•	•		•
5-Aminovaleric acid	•	•		•
5-Methyl-THF			•	•
AICAR cyclic phosphate			•	•
ATP				•
Aspartic acid	•	•		•
Benzoic acid	•			•
Carbamoyl phosphate	•	•		•
CDP-choline	•	•		•
CMP	•	•		•
Cyclic bis(3->5) dimeric GMP			•	•
dAMP	•	•		•
D-Erythrose-4-phosphate	•			•
D-Glucosamine-6-phosphate				•
Ethylmalonic acid			•	•
FAPy-adenine				•
Fructose-6-phosphate	•			•
Galactosylhydroxylysine	•			•
Glutaconic acid	•		•	•
Glycineamideribotide	•		•	•
Glycolic acid	•	•		•
GMP	•	•		•
Homocysteic acid	•	•		•
Homocysteine	•		•	•
Hypusine	•	•		•
IDP	•	•		•
IMP	•	•		•
Inosine	•	•		•
Isocitrate				•
Maltitol	•		•	•

Methylmalonic acid				•
Myo-inositol-phosphate	•	•		•
N-acetyl-glucosamine	•	•		•
NAD+	•	•		•
N-Carbamoyl-L-aspartate	•		•	•
N-Formylglycineamide ribonucleotide				•
O-Acetyl-L-serine	•	•		•
Phosphoserine	•		•	•
Spermine	•			•
Succinic acid				•
UDP	•			•
UDP-D-glucose	•	•		•
UDP-N-acetyl-glucosamine	•	•		•
UTP	•	•		•

• - Metabolite is detected in a matrix. Empty cell reports on not detected substance.

Table S2. Impacted human urine metabolites in DUS.

**Metabolite**	**Not detected in DUS**	**DUS blank Interference**
2-Dehydro-D-gluconate		•
7,8-Dihydrofolate	•	
Adenylosuccinate	•	
AMP		•
ATP	•	
Betaine aldehyde		•
Cholesterol sulfate		•
Cysteineglutathione	•	
dCDP	•	
Deoxyadenosine		•
Deoxycytidine		•
FAD	•	
Flavone		•
Fructose-1,6-bisphosphate	•	
Fumaric acid	v	•
Glucose-6-phosphate	v	
Glutathione disulfide	v	
Glutathione		•
Glyceric acid	v	•
GMP	•	
Hexadecandioic acid		•
Imidazole		•
Lactic acid		•
Leucine		•
Lipoate	•	
LPC (16:0)		•
Mevalonate-5-phosphate	•	
Myristoylcarnitine		•
NADH	•	
NADPH	•	

Nicotinic acid		•
Oleoylcarnitine		•
Oxalate		•
Palmitoylcarnitine		•
Phosphotyrosine	•	
Proline		•
Spermidine	•	
Thymidine		•
Thymine	•	
UDP-D-glucose	•	
UTP		•

• - Metabolite is not detected in DUS or is compromised with the paper carrier interference. v – results are vary from not detected to detected.

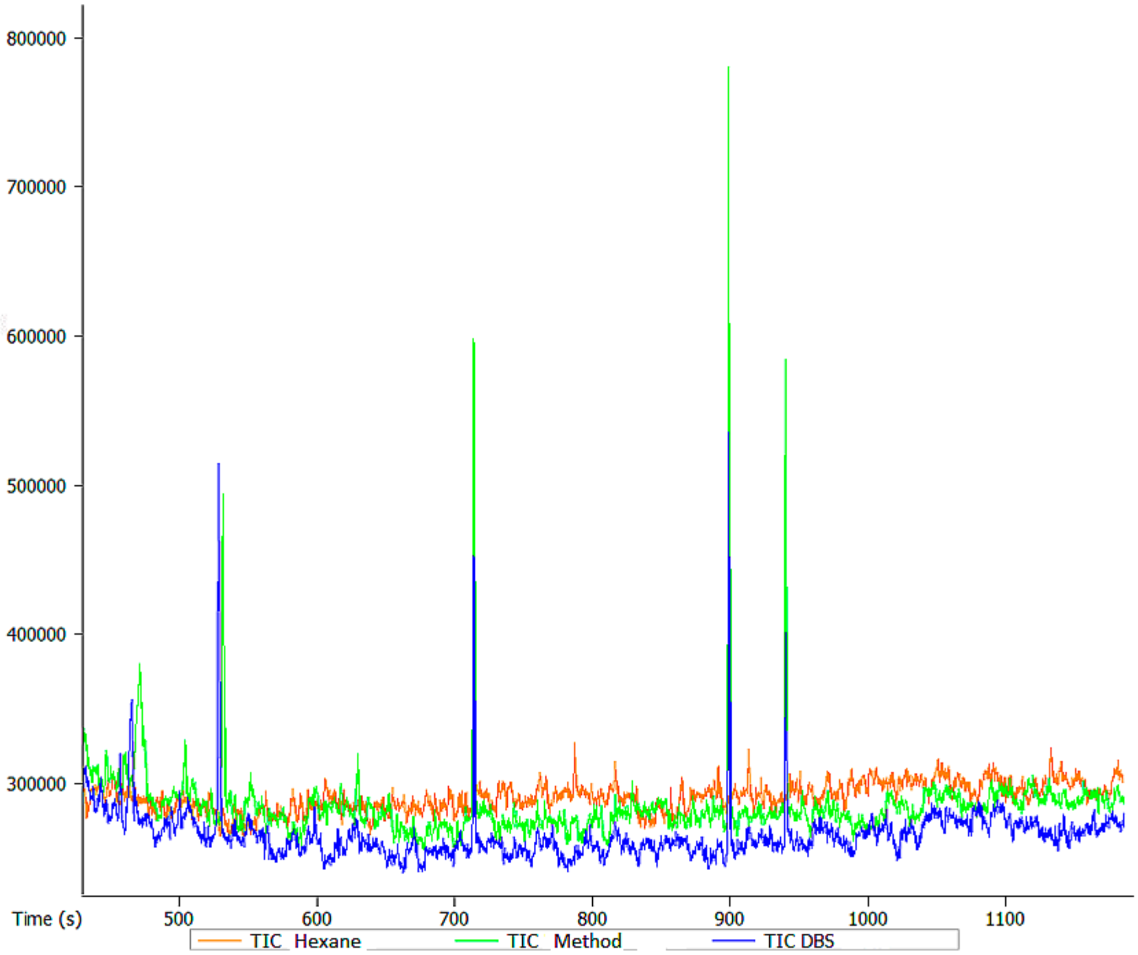

Figure S1: Overlaid GC/MS chromatograms. The following colors depict samples consequently analyzed: orange – hexane with no derivatization; green – method blank, prepared with derivatization and without actual sample; blue – DBS card derivatized extract.

Sampling and sample preparation was performed wearing gloves to avoid fatty acids transfer from the operator's skin in to the sample. Derivatization protocol included methoxymation and TMS capping at 60 °C [28]. Experiment was conducted in triplicates. Evidently admixtures were coming from derivatization procedure.

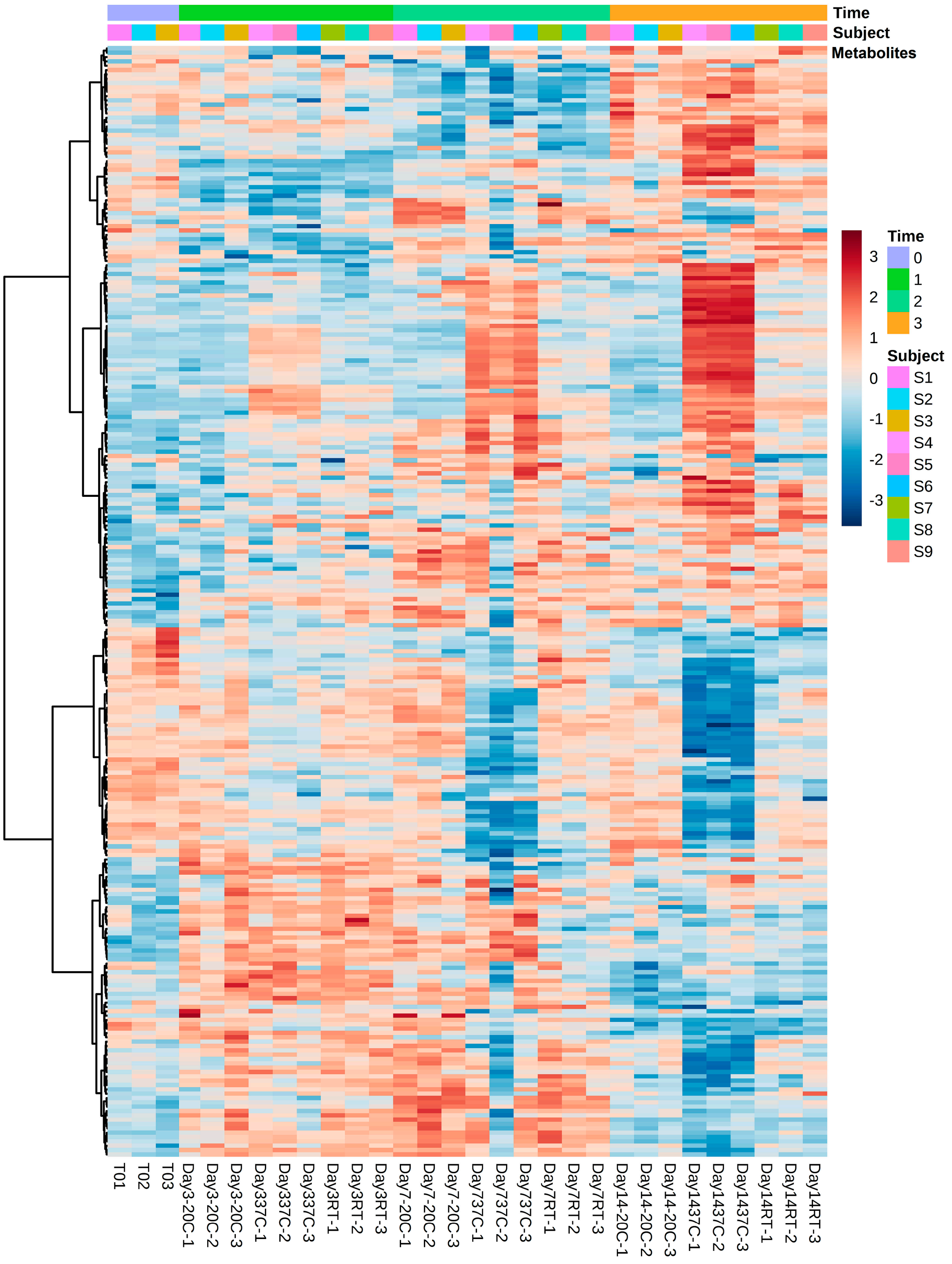

Figure S2: Hierarchical clustering analysis results of two weeks DBS storage stability study generated using MetaboAnalist 3.0 [34]. Red color represents increased metabolite levels and blue exemplifies decreased ones. Time and subject describe days and storage temperature.

Variations were observed between samples at the times T01-T03 collected on the day 0, as well as between other technical replica samples of this study. The highest level of variability observed at 37°C on Day14 of the storage conditions.
